# *Purealusbeckelorum*, a new genus and species of cleonine weevil from western Texas and eastern New Mexico (Coleoptera, Curculionidae, Lixinae, Cleonini)

**DOI:** 10.3897/zookeys.785.26674

**Published:** 2018-09-13

**Authors:** Robert S. Anderson

**Affiliations:** 1 Beaty Centre for Species Discovery, Canadian Museum of Nature, PO Box 3443, Station D, Ottawa, ON. K1P 6P4, Canada Canadian Museum of Nature Ottawa Canada

**Keywords:** Biodiversity, endemism, new species, species discovery

## Abstract

The new genus *Purealus* Anderson (type species, *Purealusbeckelorum***gen n. et sp. n.**, type locality: McKenzie Lake, Gaines County, Texas) is described to accommodate a new species of cleonine weevil from western Texas and eastern New Mexico. Habitus images and images of taxonomically significant structures are presented. *Purealus* appears to be unique within Cleonini in the distinctly tumescent and asperate base of elytral interval 3, and widely separated procoxae, two characters apparently not shared with any other world Cleonini. The species cannot be placed in either *Apleurus* or *Scaphomorphus* due to a combination of characters in part shared by each genus and the lack of characters used to define the monophyly of each genus. Coding of the character states and addition to the analysis presented in a recent comprehensive analysis of world Cleonini did not provide any significant information on phylogenetic affinities. No information on plant associations is available; most specimens have been collected walking on the ground in winter months in arid habitats.

## Introduction

During a visit to the weevil collection of retired colleague Charles W. O’Brien in Green Valley, Arizona in May of 2016 I found 5 specimens of an odd cleonine weevil collected in western Texas in October of 2004 that I was unable to identify. Further examination and comparison with other described species of North American Cleonini verified that the species was new and furthermore, questioned the generic placement of the new species which was not accommodated in any of the present North American genera *Stephanocleonus*, *Apleurus* or *Scaphomorphus*. This discovery led to a short trip to western Texas in October of 2016 during which time two additional specimens were acquired, one collected in the field at McKenzie Lake, the second collected in March of 2016 by Darren and George Pollock in Quay County, New Mexico. Since then, three additional specimens have turned up based on the collecting efforts of Darren and George Pollock, and Boris Kondratieff.

The present paper describes this new genus and species and discusses the placement of the genus with respect to *Apleurus* and *Scaphomorphus* and considering the recent overview and phylogenetic analysis of world Cleonini (Arzanov and Grebennikov 2017).

## Materials and methods

Generic and species description follows Anderson (1988). Specimens are deposited in the following collections:

**CMNC**Canadian Museum of Nature, Ottawa, Ontario, Canada; F. Génier;

**CWOB** Charles W. O’Brien collection, Green Valley, Arizona; C. W. O’Brien;

**ENMU**Eastern New Mexico University, Portales, New Mexico; D. A. Pollock.

## Taxonomic treatment

### Purealus gen. n.

Taxon classificationAnimaliaColeopteraCurculionidae

http://zoobank.org/8512E7F9-5537-4D53-942E-7A7BE835DBB8

Figures 1–3 , 4–7 , 8

#### Type species.

*Purealusbeckelorum* sp. n. by present designation.

#### Diagnosis.

Cleonini with body form moderately robust, rostrum medially longitudinally tumescent with dorsal contour in lateral view evenly rounded, procoxae widely separated, base of elytral interval 3 distinctly tumescent and asperate.

#### Description.

*Size.* Moderately large, body length 7.8–9.4 mm (exclusive of head and rostrum), moderately robust in body form. *Mouthparts.* Prementum rugose, concave medially, carinate laterally, with 2–3 large and 2–3 smaller setae on each side, a pair of long setae towards middle at apical margin. Maxillary and labial palpi not externally visible. *Rostrum.* Robust, longitudinally medially tumescent, dorsal contour in lateral view distinctly arched, rounded. Median tumescence low, not evident as distinct carina. Epistoma slightly swollen, produced anteriorly, with apical margin very slightly, very broadly, apically emarginate. Antenna with funiculus with article I subequal in length to, to slightly longer than, article II; club of four articles, apical three articles of club with placoid sensillae. *Head.* Eye elongate teardrop shaped, slightly prominent, not convex dorsally. Area behind eye with shallow irregular punctures. Upper margin of eye rounded, frons convex especially at base of median rostral tumescence. *Vestiture.* Dorsum with suberect or erect vestiture short and sparse; with simple very elongate-narrow recumbent white hair-like scales of variable size and density. *Prothorax.* Dorsal surface of pronotum coarsely punctate. Pronotum with median basal area deeply impressed towards basal margin; anterolateral margin, behind eyes, with moderately developed rounded postocular lobe; postocular vibrissae short, of equal length; disk with elongate white larger moderately dense scales present in lateral sinuate stripe, variously larger and denser along lateral margins; median area largely black in color, underlying black cuticle not obscured by any scales, subglabrous. Prosternum with very slight impression anterolaterad of each procoxal cavity; without any swelling immediately anterior to each prosternal impression; procoxae widely separated by about one-half width of coxa by globular posterior process of prosternum. *Legs.* Foretarsus moderately broad, articles II and III more or less subequal in length, at most only slightly longer than broad; article I longer than articles II or III; article III deeply bilobed. Meso- and metatarsus very slightly more elongate-narrow, article II slightly to distinctly longer than article III; article I distinctly longer than articles II or III; article III deeply bilobed. Ventral tarsal pilosity reduced to some extent (not covering entire ventral surface of a tarsal article) to almost lacking entirely from at least more basal tarsal articles (especially of metatarsus). Claws connate from near base to through basal one-third, slightly to markedly divergent. Foretibia with inner margin lacking denticles; near apex with second spur not developed. Tibia with corbel ridge rounded. *Hind wings.* Absent. *Elytra.* Intervals, except humerus and bases of intervals 3, 5, 7, and 9 flat, bases of intervals 3, 5, 7, and 9 slightly to markedly swollen, asperate (especially that of interval 3 which is tumescent). Striae confused, punctate. Humeri rounded. Scales variously condensed in patches of larger, denser white scales. *Abdomen.* Ventral surface almost uniformly covered with fine dense white hair-like scales. Abdominal sternum VII in males with apical margin at middle simple, without small dorsally directed median tooth. *Genitalia.* Female. Abdominal sternum VIII lacking basal arm (spiculum gastrale). Gonocoxite II elongate triangular in form, apex not prolonged into lobe; stylus present, moderately large in size; apical. Spermathecal gland oval. Symbiont pouches elongate-oval lying adjacent to vagina, composed of 10–12 rings, joined to vagina near apex. Male. Abdominal sternum VIII with interior angle of each sclerite lacking basal projection. Aedeagus moderately robust, in lateral view more or less evenly arcuate throughout length; apex not spatulate, somewhat acuminate. Internal sac not everted. Apical sclerite complex not evident.

#### Derivation of generic name.

The generic name *Purealus* is derived as an anagram of *Apleurus*. Gender masculine.

#### Natural history.

Five specimens of this species were collected in October 2004 at McKenzie Lake, Gaines County, Texas by tiger beetle enthusiast Dave Brzoska while hunting tiger beetles. The exact circumstances of capture are not known but the specimens were likely walking around on the ground in areas inhabited by tiger beetles. One specimen was collected dead by me in fine debris and other insects washed up along the northern shore of McKenzie Lake in October 2016. Another three specimens were collected in Lea and Quay Counties, New Mexico, walking on the ground. During my trip to McKenzie Lake numerous species of shrubs and herbs were searched for this species without success. Searches of tiger beetle habitats such as open sandy flats, open alkaline areas around the lake, and dirt roadways also yielded no specimens.

#### Comments.

**Figures 1–3.**
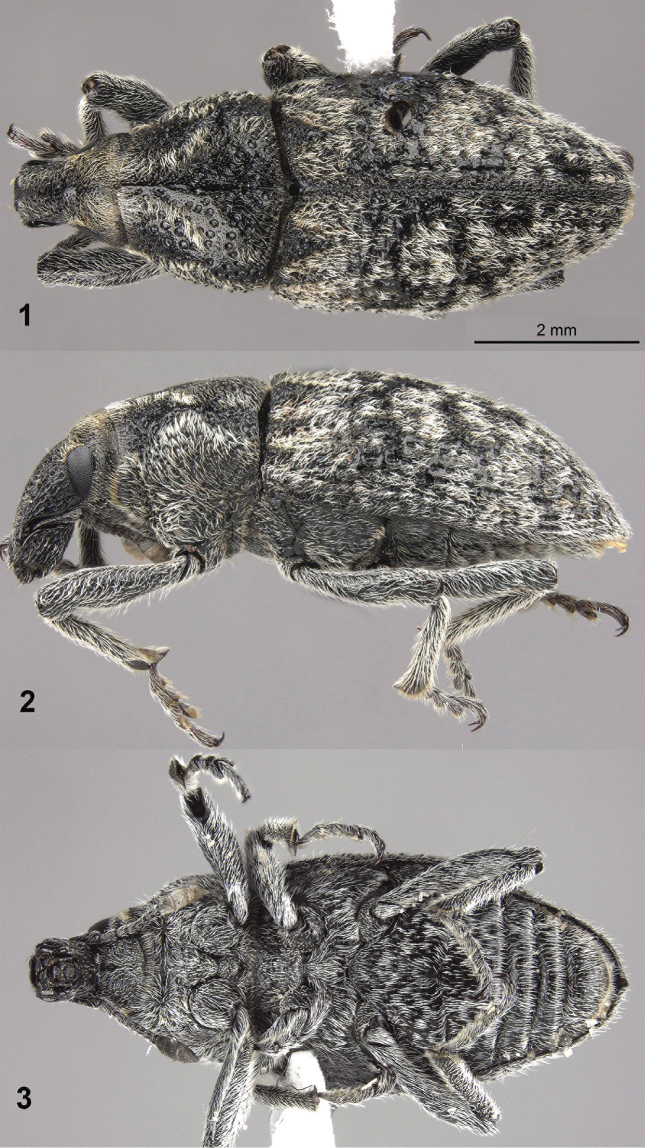
*Purealusbeckelorum*. **1** Dorsal habitus **2** Lateral habitus **3** Ventral habitus.

The robust form, robust, medially longitudinally tumescent rostrum with dorsal contour in lateral view evenly rounded (Fig. 2), but especially the widely separated procoxae (Fig. 3) and the distinctly tumescent and asperate base of elytral interval 3 (Figs 1–2), will readily distinguish this genus among all Cleonini. Such widely separated procoxae are apparently not known in any other world Cleonini, all of which have the procoxae contiguous. The genus combines characters of the North American genera *Apleurus* Chevrolat and *Scaphomorphus* Motschulsky with the rounded tibial flange and rounded postocular lobes of *Scaphomorphus* but the robust rostrum, reduced ventral tarsal vestiture and more robust form of an *Apleurus*. Characters used to recognize the monophyly of *Scaphomorphus* (shiny glabrous median tubercle at base of abdominal sternum VII in female) and various groups of *Apleurus* (blunt tooth at apical margin of abdominal sternum VII in male, swellings anterior to front coxae, apically rounded epistoma) are not present in *Purealus*.

### Purealus beckelorum sp. n.

Taxon classificationAnimaliaColeopteraCurculionidae

http://zoobank.org/04568E7E-09BB-4EB4-9115-61B760974C93

Figures 1–3 , 4–7 , 8

#### Diagnosis.

Rostrum dorsally medially longitudinally tumescent, not distinctly carinate; elytra with humerus rounded, bases of intervals 3, 5, 7, and 9 elevated and variously convex, especially base of interval 3 which is markedly tumescent and distinctly asperate.

#### Description.

*Size.* Length, male, 7.8–9.4 mm; female, 8.5–9.0 mm. Width, male, 3.5–4.2 mm; female, 3.5–4.2 mm. *Head*. Eye very slightly prominent and very slightly convex in dorsal view. Frons and vertex with moderately dense, moderately large, deep punctures. Frons also with sparse, short suberect golden or white hair-like scales densest immediately above eyes. Area immediately behind posterior margin of eye with small shallow indistinct punctures. Area above eyes not elevated above rest of frons (eyes not browed in anterior view). Width of frons subequal to width at apex of rostrum. Rostrum robust (width at apex 0.76–0.82 times length). Median longitudinal tumescence present, broad, low, not carinate. Dorsal and lateral punctation moderately dense, moderately deep. Dorsally, excluding epistoma, with moderately dense, short to long, recumbent elongate white hair-like scales. In lateral view with apical portion flat to very slightly declivous from point of antennal insertion to apex. Epistoma with apical margin very slightly, very broadly emarginate at middle. *Pronotum*. In dorsal view with lateral margins subparallel to slightly convergent from base to apical one-quarter; constricted at oblique angle anterior to apical one-quarter; apical one-quarter and base subequal in width; without distinct lateral tubercles. Dorsal and lateral punctation large, moderately dense, deep; punctures less distinct, sparser and smaller on flanks. Scales white, elongate-narrow, appressed, lacking medially from disk and dorsally from flanks, dense laterally in sinuate line and at lateral margins. Median carina slightly developed in anterior one-half or lacking. Dorsally with dense very short fine suberect white hair-like scales each situated within large puncture. Anterolateral margin with postocular rounded lobe distinct. *Elytra*. Very robust in general form (width at midlength 0.65–0.69 times length. In dorsal view with lateral margins arcuate throughout length. Humerus rounded, indistinct. Dorsally with bases of intervals 3, 5, 7, and 9 elevated and variously convex, especially base of interval 3 which is markedly tumescent and distinctly asperate, otherwise remainder of all intervals flat. Scales white, various in density and size; elongate-narrow, hair-like, pattern various but with larger and denser scales condensed in variously arranged patches. Wings absent. Legs. Ventral pilose vestiture of foretarsus present as fine elongate lines on apical one-half of articles I and II, and as oval pads covering apical one-half of article III; of mesotarsus present as minute apical tufts on article I, as apical small fine line on article II, and as very small oval apical pads on article III; of metatarsus present as minute apical tufts on articles I and II, and as very small oval apical tuft on article III. *Abdomen*. Ventral surface with uniformly moderately dense, elongate-fine recumbent white hair-like scales. *Genitalia. Female*. Abdominal sternum VIII with lateral arms narrow, divergent basally, markedly inwardly arcuate at about midlength then strongly convergent to apex. Gonocoxite II with stylus moderately large, apical in position. *Male*. Abdominal sternum VIII with paired sclerite with inner apices lacking ventral projections. Aedeagus elongate-narrow, in lateral view thickest near base; in ventral view with lateral margins slightly convergent to near apex, then strongly convergent subapically to acuminate tip. Internal sac not everted.

**Figures 4–7.**
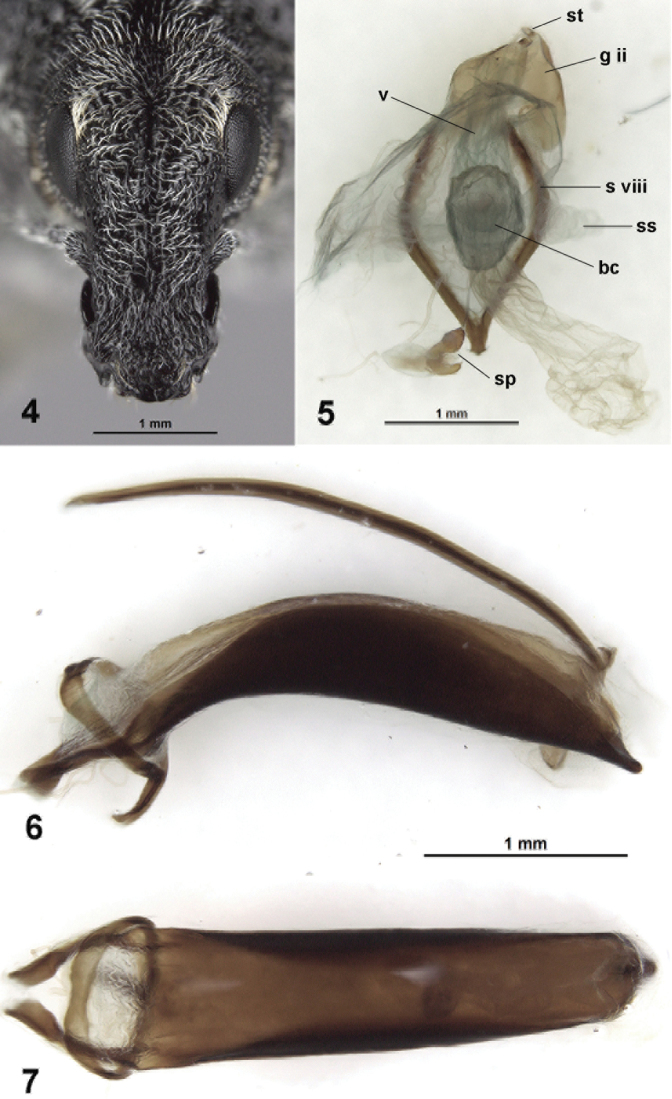
*Purealusbeckelorum*. **4** Head, dorsal view **5** Female genitalia, dorsal view **6** Aedeagus, lateral view **7** Aedeagus, dorsal view. Abbreviations: bc, bursa copulatrix; g ii, gonocoxite ii; s viii, sternite viii; sp, spermatheca; ss, symbiont sac; st, stylus; v, vagina.

**Figure 8.**
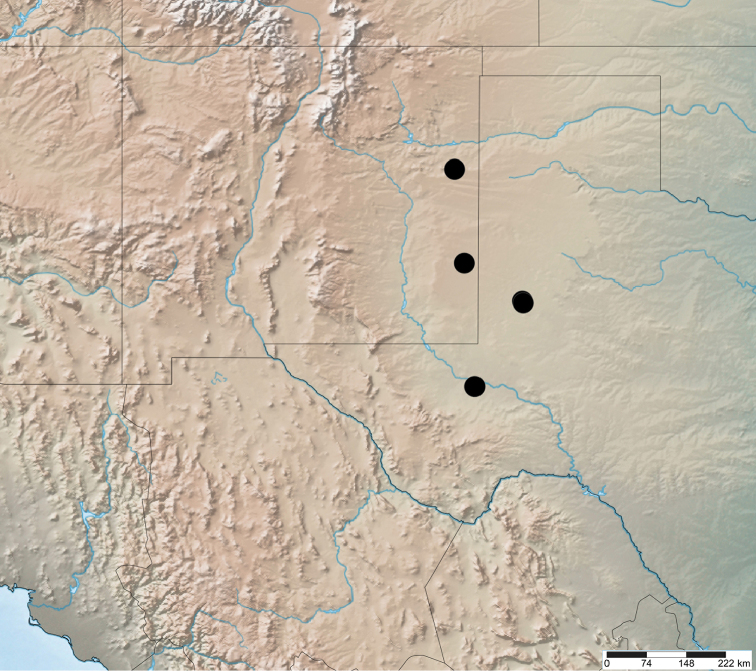
*Purealusbeckelorum*, distribution.

#### Specimens examined.

Holotype male, labelled Texas: Gaines County, McKenzie Lake, N.E. shore, 32.72536 -102.32003, 929m, 19.x.2016, R.S. Anderson, beach washup, 2016-205 (CMNC). Paratypes, Texas: Gaines County, Highway 380, McKenzie Draw (Lake), 920m, 32 41.5’N 102 18.4’W, 19.x.2004, D. Brzoska (1 male, 4 females, CMNC, CWOB). Pecos County, Coyanosa Draw, [31.2882 -103.1216], Rte. 285, 11.iii.1990, B. Kondratieff & F. Welch (1 male, CMNC). New Mexico: Lea County, County Road 164 (Ranger Lake Road), 1.3 mi. E. Highway 206, 4007’, 33.358278 -103.295226, 25.xi.2016, D. & G. Pollock (1 female, CMNC). Quay County, Highway 278, ca. 13.8 mi. S.W. San Jon, Apache Canyon, 4600’, 34.934684 -103.460314, 27.iii.2016, D. & G. Pollock (1 female, ENMU); same except 11.xii.2016 (1 female, CMNC).

#### Derivation of species name.

The species is named after the Beckel family of Vancouver, Canada. William Edwin and Dorothy (née Brown) Beckel, parents of Canadian Museum of Nature President, Margaret Beckel, graciously funded the field work in October of 2016 in an attempt to collect additional specimens of the species and to discover aspects of its biology and host plants. William Beckel was president of Carleton University (Ottawa, Ontario) for the years 1979–1989 and before that President of the University of Lethbridge (Lethbridge, Alberta). Previously William Beckel was the Head of the Entomology Lab and Dorothy Beckel was the Head of the Botany Lab at the Northern Defense Research Laboratory at Fort Churchill, Manitoba in the early 1950s.

#### Natural history.

Specimens were collected in the months of October, November, December, and March. It is possible that the species is winter active and as such has not been discovered previously. One specimen was collected dead in beach wash-up and three other specimens were collected walking on the ground. There are no plant associations known.

#### Comments.

This species bears a superficial resemblance to *Scaphomorphuscanescens* (LeConte), a species found in the same general area. The large, asperate swellings at the base of elytral interval 3 and widely separated procoxae are distinctive and apparently not known in other world Cleonini. Despite examination of thousands of specimens from almost all major museums, no specimens of this species were seen by Anderson (1988).

This addition prior to couplet 1 in the key to North American Lixinae (Anderson 2002) or North American Cleonini (Anderson 1988) will accommodate *Purealus* (references are to figures in this paper):

**Table:** 

1'	Procoxae widely separated by about one-half width of procoxa by globular posterior prosternal process (fig. 3). Elytra with base of interval 3 tumescent and asperate (figs 1–2), humeri absent (rounded) (fig. 1)	***Purealus* Anderson**
–	Procoxae contiguous. Elytra with base of interval 3 variously swollen or not, not asperate, humeri absent or present	**1**

## Phylogenetic considerations

As noted, this genus is characterized by the distinctly tumescent and asperate base of elytral interval 3 and the widely separated procoxae, two characters apparently not shared with any other world Cleonini. These are both significant characters, particularly the widely separated procoxae, a character used in many beetle groups as of higher taxonomic significance. With regard to the North American fauna, *Purealusbeckelorum* cannot be accommodated in the genera *Apleurus* or *Scaphomorphus* due to a combination of characters in part shared by each genus and by the lack of characters used to define the monophyly of each genus (or major part thereof). For *Scaphomorphus*, this is the presence of a shiny glabrous median tubercle at the base of abdominal sternum VII in the female, and for various groups of *Apleurus*, the presence of a blunt tooth at the apical margin of abdominal sternum VII in the male, prosternal swellings anterior to the front coxae, and an apically rounded epistoma. *Purealus* has reduced ventral tarsal vestiture on the middle and hind legs, and rounded humeri, character states each shared with some *Apleurus* and *Scaphomorphus*, and has distinct postocular lobes and an apical stylus on gonocoxite II of the female, character states shared with all or most *Scaphomorphus* but not *Apleurus*.

The possible phylogenetic relationship of the genus with respect to the world fauna was investigated following the character analysis of world Cleonini presented by Arzanov and Grebennikov (2017). They scored adult morphological characters for 79 of a total of 96 extant genus-group Cleonini taxa considered valid to date. Their resulting matrix contained 121 parsimoniously informative characters scored for 145 ingroup (Cleonini) and 29 outgroup terminals. Neither the extent of procoxal separation or the condition of the base of elytral interval 3 are coded as distinct characters likely because states of these two characters in *Purealus* appear to be unique. Here, I code the 121 characters listed in Arzanov and Grebennikov (2017) (not relisted here) for *Purealusbeckelorum*. The string of 121 character scores is as follows:

11101100100010001011000000??????100001100010100110101000000000000001010110101011110

?????????????????001000000000010000000.

No attempt was made to inflate the lobes of the internal sac of the aedeagus as done by Anderson (1988) and Arzanov and Grebennikov (2017) and mouthparts were not dissected (hence the “?” in the string above).

The results of the new analysis places *Purealusbeckelorum* as sister to *Glebiusconfluens* (Fahraeus) with negligibly small statistical support of 8% (see Arzanov and Grebennikov [2017], figs. 8–9 for placement of *Glebiusconfluens*). However, as discussed by Arzanov and Grebennikov (2017) their results showed that relationships within the Cleonini remain largely unresolved with either 47 (Bayesian inference) or 37 (maximum parsimony) branches radiating from the tribe’s most recent common ancestor so this putative sister-group relationship of *Purealus* is almost meaningless. Specimens of *Glebiusconfluens* (CMNC) were examined but show no shared significant character states with *Purealusbeckelorum*. Phylogenetic relationships of the new genus are thus best considered unknown and the genus another member of the unresolved basal comb of Arzanov and Grebennikov (2017).

## Supplementary Material

XML Treatment for Purealus

XML Treatment for Purealus beckelorum
